# Resolutions of Thanks

**Published:** 1867-06

**Authors:** 


RESOLUTIONS OF THANKS.

Dr. Atlee offered the following:—

*Resolved*, That the thanks of this Association be presented to the Committee of Arrangements, to the corporate authorities of Cincinnati, to his Honor, Mayor Wilstach, Mr. Geo. H. Pendleton, Mr. Larz Anderson, Dr. Geo. Mendenhall, and the citizens generally, for the cordial and generous hospitalities extended to the members of the Association during their present session.

Dr. Levick, of Philadelphia, remarked upon the resolution, saying that no body of men were ever before received with more refined and generous hospitality, and he had been sorry to hear certain remarks deprecating this manifestation of the social element of these meetings. It was an important element in promoting harmony, and, therefore, the dignity of the profession. The resolution was adopted by a unanimous vote.

A resolution of thanks to the following named railroads, for commutation of fares, was also passed:—Baltimore & Ohio, Marietta, Little Miami, Columbus & Cleveland, Cincinnati, Hamilton & Dayton, Dayton & Michigan, Cincinnati, Indianapolis & Lafayette, Covington & Lexington, Chicago & Great Eastern.

Dr. Cox remarked, that in presenting the matter in person to the President of the Baltimore & Ohio, that large-minded and liberal-hearted man said, that if a body of politicians, or almost any other class of men, had applied for such a favor, he would most likely have refused them; but that, regarding us as benefactors of the race, he felt happy to contribute what he could to promote our interests and comforts.

Dr. Hughes, of Iowa, offered a resolution of thanks to the press of this city, for their full and impartial reports of the proceedings of the Association.

